# Pic-Producing *Escherichia coli* Induces High Production of Proinflammatory Mediators by the Host Leading to Death by Sepsis

**DOI:** 10.3390/ijms21062068

**Published:** 2020-03-18

**Authors:** Itaynara L. Dutra, Lorena G. Araújo, Raissa G. Assunção, Yago A. Lima, Johnny R. Nascimento, André A. M. Vale, Patrícia C. S. Alves, Liana O. Trovão, Ana Carolina M. Santos, Rosa M. Silva, Lucilene A. Silva, Márcia C. G. Maciel, Eduardo M. de Sousa, Waldir P. Elias, Flávia R. F. Nascimento, Afonso G. Abreu

**Affiliations:** 1 Laboratório de Patogenicidade Microbiana, Universidade Ceuma, São Luís 65075-120, Brazil; itaynaradutra@hotmail.com (I.L.D.); araujo.lorenabio@gmail.com (L.G.A.); raissa_guara@hotmail.com (R.G.A.); 2 Programa de Pós-Graduação em Ciências da Saúde, Universidade Federal do Maranhão, São Luís 65080-805, Brazil; john_nyramos@yahoo.com.br (J.R.N.); andre_amvale@hotmail.com (A.A.M.V.); patriciacosta88@bol.com.br (P.C.S.A.); lianatrovao@hotmail.com (L.O.T.); lucileneamorimsilva@yahoo.com.br (L.A.S.); macielmcg@gmail.com (M.C.G.M.); edmsousa@hotmail.com (E.M.d.S.); nascimentofrf@yahoo.com.br (F.R.F.N.); 3 Programa de Pós-Graduação em Biologia Microbiana, Universidade Ceuma, São Luís 65075-120, Brazil; 4 Laboratório de Imunofisiologia, Universidade Federal do Maranhão, São Luís 65080-805, Brazil; yagohh_center@hotmail.com; 5 Departamento de Microbiologia, Imunologia e Parasitologia, Escola Paulista de Medicina, Universidade Federal de São Paulo, São Paulo 04023-062, Brazil; carolina.mello@unifesp.br (A.C.M.S.); rosa.unifesp@gmail.com (R.M.S.); 6 Departamento de Biologia Celular, Universidade de Brasília, Brasília 70878-040, Brazil; 7 Laboratório de Bacteriologia, Instituto Butantan, São Paulo 05503-900, Brazil; waldir.elias@butantan.gov.br

**Keywords:** *Escherichia coli*, Pic, serine protease, virulence, sepsis

## Abstract

*Escherichia coli* is an important pathogen responsible for a variety of diseases. We have recently shown that Pic, a serine protease secreted by *E. coli*, mediates immune evasion by the direct cleavage of complement molecules. The aim of this study was to investigate the action of a Pic-producing bacteria in a murine model of sepsis. Mice were infected with Pic-producing *E. coli* (F5) or F5∆*pic* mutant. Animal survival was monitored for five days, and a subset of mice was euthanized after 12 h for sample acquisition. The inoculation of Pic-producing bacteria induced 100% death within 24 h. The colony forming units count in the organs was significantly higher in F5. Hematological analysis showed a decrease of total leukocytes. Nitric oxide and cytokines were detected in serum, as well as on peritoneal lavage of the F5 group in higher levels than those detected in the other groups. In addition, immunophenotyping showed a decrease of activated lymphocytes and macrophages in the F5 group. Therefore, Pic represents an important virulence factor, allowing the survival of the bacterium in the bloodstream and several organs, as well as inducing a high production of proinflammatory mediators by the host, and concomitantly a cellular immunosuppression, leading to sepsis and death.

## 1. Introduction

Sepsis is known as a life-threatening organ dysfunction resulting from an overwhelming immune response triggered by invading pathogens [1]. This process begins when pathogen-associated molecular patterns (PAMPs) are recognized by pattern recognition receptors (PRRs) from host immune cells, which results in several protein phosphorylation cytoplasmic reactions following transcriptions of genes encoding inflammatory mediators, such as cytokines, chemokines, and nitric oxide synthase [2]. The over activation of the immune response causes cardiovascular, hemodynamic, neurological, metabolic alterations, and harmful activation of several plasmatic proteins, such as the coagulation cascade and complement system, which contribute to the worsening of the patient’s clinical condition, resulting in impairment of the functionality of several organs and systemic failure, leading to death in some cases [3,4,5].

Sepsis represents a worldwide public health concern due to the high mortality rate and its difficult and expensive treatment [6]. Comparing the mortality of hospitalized patients in intensive care units (ICUs) from different countries, a high mortality was observed in Brazil (56.1%) followed by Argentina (46.6%), United States of America (33%), Canada (30.3%), and Australia (22%) [7]. In the United States of America, the incidence of sepsis is estimated at 300 cases per 100,000 inhabitants and the costs of treating these patients reach 14 billion dollars [8]. In Brazil, the costs resulting from these hospitalizations, in 2015 alone, were approximately R$ 400 million [9].

Both Gram-positive and Gram-negative bacteria can trigger the sepsis process, with *Escherichia coli* being among the major Gram-negative causes of sepsis [8]. *E. coli* is a bacterium widely distributed in several environments and commonly found in the large intestine of humans and warm-blooded animals, without causing disease in healthy individuals [10]. However, some strains are able of causing intestinal disorders such as diarrhea, which is a serious public health problem worldwide, as well as causing morbidity and mortality among children in developing countries [11]. Pathogenic strains of *E. coli* are also reported as an etiologic agent of urinary tract, central nervous system, and bloodstream infections [12].

Bloodstream infections have been related to the various pathovars of *E. coli* [13,14,15]. The strains that cause bacteremia have several virulence factors, including those involved with adhesion, immune system evasion, acquisition of iron, toxins production, and resistance to bactericidal action of the serum [16,17]. The presence of these factors can ensure the persistence of these bacteria in the blood and dissemination through the organism and can consequently trigger a severe inflammatory response resulting in sepsis [18,19].

Among the virulence factors that enable *E. coli* to evade the immune system is the protein involved in colonization (Pic). It is a serine protease of 116 kDa produced by some bacteria from the *Enterobacteriaceae* family, such as *E. coli* [20,21,22,23], *Shigella flexneri* [24], and *Citrobacter rodentium* [25], and secreted via the type V secretion pathway [26,27]. Several biological roles for Pic were described, including mucinolytic activity, hemagglutination, coagulation cascade factor V degradation, and leukocyte surface glycoprotein cleavage [20,21,28,29]. In addition, Pic promotes both intestinal colonization of mice and rabbits [20,30] and resistance to the serum by direct cleavage of key molecules belonging to the three pathways of the complement system cascade, such as C2, C3, C3b, C4, and C4b. It is important to mention that Pic is able to degrade human complement proteins as purified molecules or in the context of normal human serum [31]. In addition, Pic has been associated with acute and persistent diarrhea [32,33,34,35,36]. In this way, Pic-producing pathogens are able to destroy the protective barrier of the intestinal epithelium causing bacterial persistence, invasion, migration to extra-intestinal sites, and some of them have the capacity to reach the bloodstream, causing bacteremia and sepsis [18].

Since Pic is able to degrade important biological substrates, the aim of this work was to evaluate the ability of the serine protease Pic produced by *E. coli* to induce lethal sepsis in animals submitted to intraperitoneal inoculation of bacteria.

## 2. Results

### 2.1. Pic-Producing E. coli (F5) Induces Lethal Sepsis in Mice

**Figure 1:**
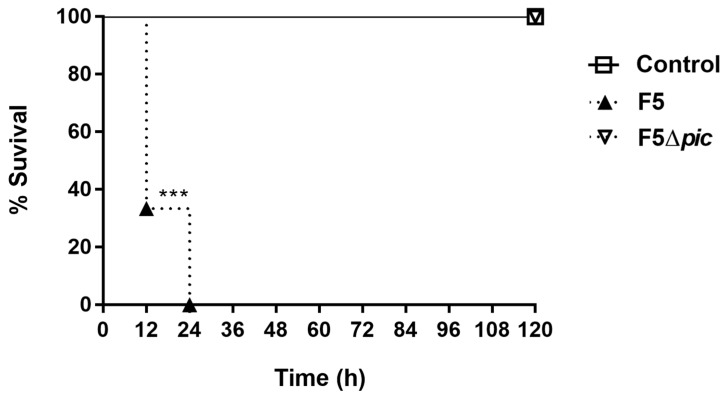
Survival curve of animals submitted to intraperitoneal inoculation of bacteria or apyrogenic water. The animals were distributed into three groups (6 animals/group). The animals in the control group received injections containing apyrogenic water. The remaining groups received Pic-producing *E. coli* (F5) or the Pic not producing mutant (F5Δ*pic*) suspensions. Thereafter, the survival was assessed for 5 days at 12/12 h post-infection intervals. Data were analyzed by log-rank statistical test. *** *p* < 0.0001 as compared with the other groups.

Intraperitoneal inoculation of F5 induced the death of approximately 66% of the animals within 12 h after infection. The remaining animals died within 24 h after bacterial inoculation, reflecting 100% mortality within 24 h after infection. However, there was no death in the F5Δ*pic* group, suggesting that the death is related to the presence of Pic (Figure 1).

### 2.2. Pic is Essential for the Permanence of the Bacteria in the Bloodstream

**Figure 2:**
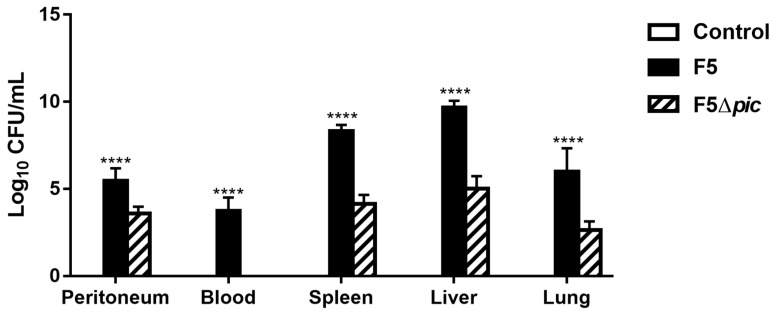
CFU count from peritoneal lavage, blood, spleen, liver, and lung from animals submitted to intraperitoneal inoculation of bacteria or apyrogenic water. The animals were distributed into three groups (6 animals/group). The animals in the control group received injections containing apyrogenic water. The remaining groups received Pic-producing *E. coli* (F5) or the Pic not producing mutant (F5Δ*pic*) suspensions. After 12 h the animals were euthanized. For evaluation of CFU, aliquots of blood and peritoneal fluid were diluted in sterile PBS and serial dilutions were seeded on MacConkey agar. To evaluate the presence of bacteria in the organs, parts of them were weighed, macerated, and dilutions were seeded on MacConkey agar. The CFUs were counted after overnight incubation at 37 °C. The results are expressed as Log of CFU/mL and represent the mean ± S.D. Data were analyzed using ANOVA, followed by Tukey’s multiple test. **** *p* < 0.0001 as compared with the other groups.

CFU counts were positive in both bacterial groups (F5 and F5Δ*pic*) on the peritoneal lavage, spleen, liver, and lung, but the number of bacteria was inferior in the F5Δ*pic* as compared with the F5 group. However, in the blood, CFU was detected only in the F5 group, suggesting that the presence of Pic is essential to maintenance in the bloodstream (Figure 2).

### 2.3. Infection Induces Histological Changes in the Organs of Animals

**Figure A1:**
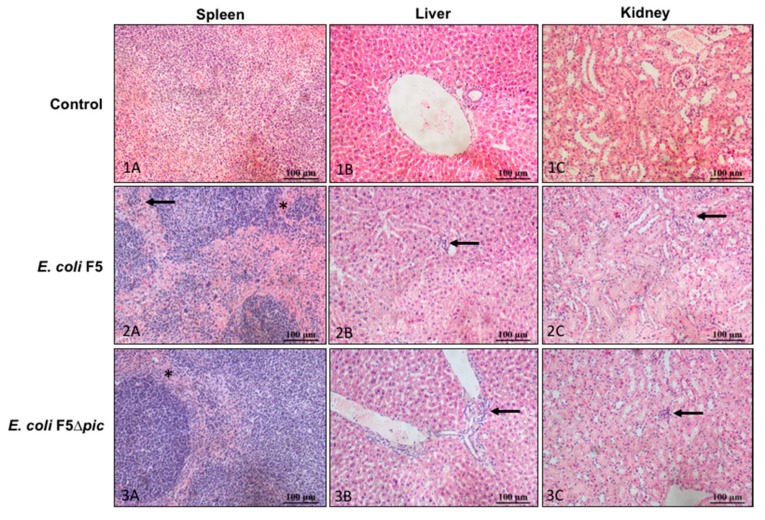
Histopathological evaluation of spleen, liver, and kidneys from animals submitted to intraperitoneal inoculation of bacteria. Twelve hours after peritoneal inoculation of bacteria, the liver, spleen, and kidney of the animals were removed, weighed, and fixed in formaldehyde, dehydrated in alcohol and embedded in paraffin. Histopathological analysis was performed using an optical microscope. Control: (**1A**) spleen, (**1B**) liver, (**1C**) kidney; *E. coli* F5: (**2A**) spleen, (**2B**) liver, (**2C**) kidney; *E. coli* F5Δ*pic*: (**3A**) spleen, (**3B**) liver, (**3C**) kidney. Asterisks indicate hemorrhagic area and arrows indicate inflammatory infiltrate.

The animals of groups F5 and F5Δ*pic* presented histopathological changes characteristic of inflammation in the spleen, liver, and kidneys. The presence of hemorrhage, cellular infiltrate, and edema in these organs was observed, but the presence of necrosis was not evidence. However, such histopathological changes did not differ statistically between the F5 and F5Δ*pic* groups (Table 1 and Figure A1 in Appendix A).

### 2.4. Pic-Producing E. coli Promotes Decrease of leukocytes in the Blood, but Does Not Induce Alterations in the Leukocytes Total Number in Bone Marrow Cells, Spleen, and Peritoneal Cavity

**Figure 3:**
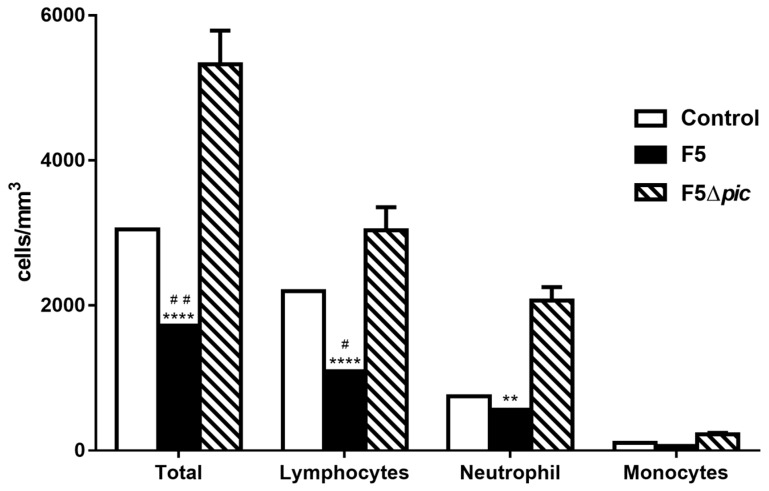
Differential blood cell counts from animals submitted to intraperitoneal inoculation of bacteria or apyrogenic water. Twelve hours after induction of sepsis by peritoneal inoculation of bacteria, the animals already anesthetized were submitted to retro-orbital puncture for blood collection for total and differential cell counts. The results represent the mean ± S.D. of 6 animals per group. Data were analyzed by Kruskal–Wallis test. ^#^ *p* < 0.05 as compared with the control group. ^##^ *p* < 0.01 as compared with the control group. ** *p* < 0.01 as compared with the F5Δ*pic* group. **** *p* < 0.0001 as compared with the F5Δ*pic* group.

On the one hand, the blood count showed a decrease of leucocytes in the blood of the animals of the F5 group as compared with the other groups (Figure 3). It occurred due to a reduction of lymphocytes which decreased as compared with the control group, while the number of neutrophils did not differ between these same groups. On the other hand, the F5Δ*pic* group presented an increase in the total number of leukocytes as compared with the control group, attributed mainly to an increase in the number of neutrophils (Figure 3).

**Figure A2:**
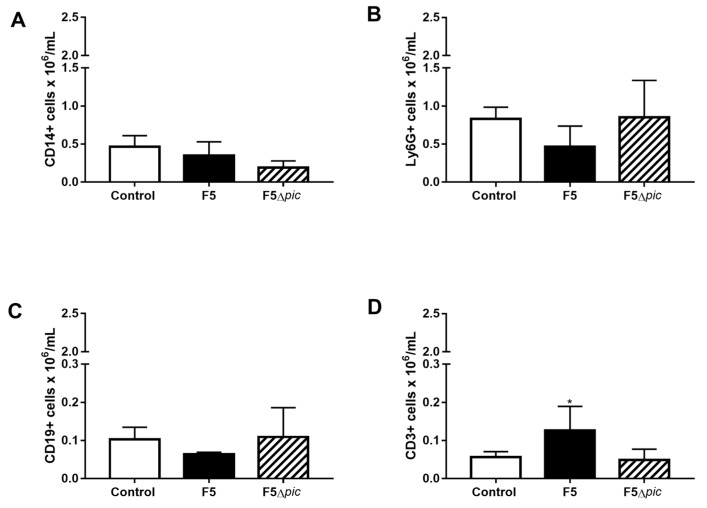
Peritoneal cell counting of animals inoculated with bacteria. The peritoneal cells of the animals were aseptically collected from the lavage of the peritoneal cavity. After cell counts of the peritoneal cavity, 10^6^ cells/mL were resuspended in specific buffer, transferred to a 96-well U-bottom plate, labeled with specific antibodies, and incubated at 4 °C for 15 min. (**A**) Number of macrophages; (**B**) number of neutrophils; (**C**) number of B lymphocytes; (**D**) number of T lymphocytes. The results represent the mean ± S.D. of 6 animals per group. Data were analyzed using ANOVA, followed by Tukey’s multiple test. * *p* < 0.05 as compared with the F5Δ*pic* group.

The total number for bone marrow cells, splenocytes, and peritoneal cells (Table 2) was similar between the groups. The number of macrophages (Figure A2A), neutrophils (Figure A2B), and B lymphocytes (Figure A2C) was not altered in the peritoneum cavity. However, the number of T lymphocytes was elevated in the F5 group (Figure A2D).

### 2.5. E. coli F5 Compromises the Expression of Costimulatory Molecules and Affects the Expression of iNOS by Leukocytes in the Peritoneal Cavity

**Figure 4:**
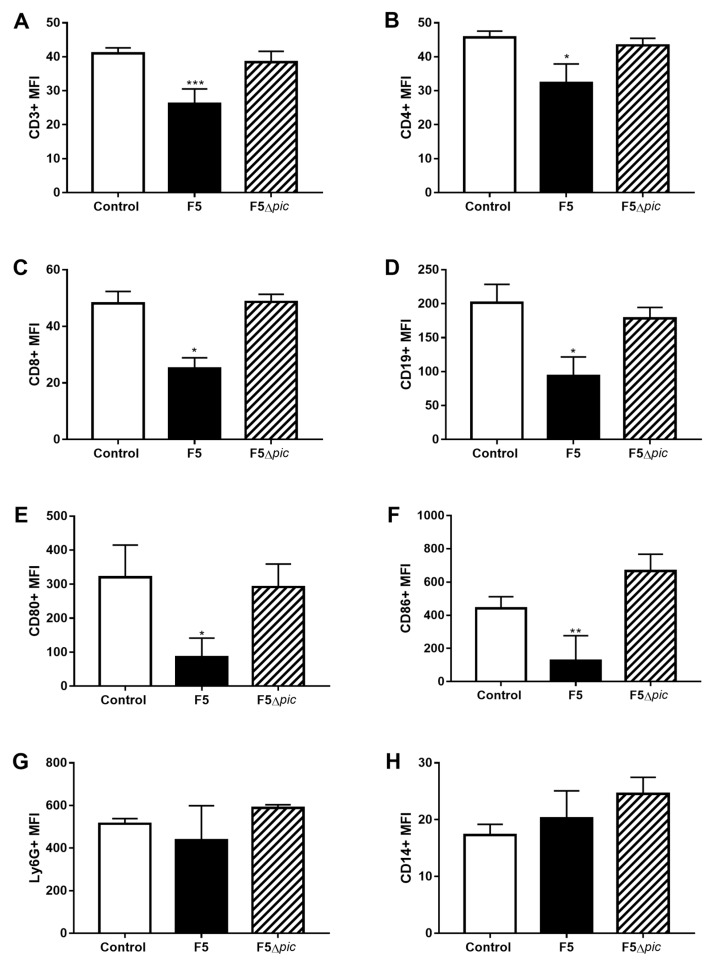
Median fluorescence intensity (MFI) of the costimulatory molecules in leukocytes from the peritoneal cavity. After cell counts of the peritoneal cavity, 10^6^ cells/mL were suspended in specific buffer, transferred to a 96-well U-bottom plate, labeled with specific antibodies, and incubated at 4 °C for 15 min. The antibodies used were anti-Ly6G, anti-CD3, anti-CD4, anti-CD8, anti-CD19, anti-CD14, anti-CD80, and anti-CD86. (**A**) Median fluorescence intensity of CD3; (**B**) median fluorescence intensity of CD4; (**C**) median fluorescence intensity of CD8; (**D**) median fluorescence intensity of CD19; (**E**) median fluorescence intensity of CD80; (**F**) median fluorescence intensity of CD86; (**G**) median fluorescence intensity of Ly6G; (**H**) median fluorescence intensity of CD14. The results represent the mean ± S.D. of 6 animals per group. Data were analyzed using ANOVA. * *p* < 0.05 as compared with the F5Δ*pic* group, ** *p* < 0.01 as compared with the other groups, *** *p* < 0.001 as compared with the F5Δ*pic* group.

Although there was no difference in the leukocyte subtypes, the peritoneal leukocytes from the F5 group presented low expression of some cell surface molecules, including receptors and costimulatory. There was a lower expression of CD3 (Figure 4A), CD4 (Figure 4B), CD8 (Figure 4C), and CD19 (Figure 4D), whereas in macrophages, a low expression of costimulatory molecules was observed, such as CD80 (Figure 4E) and CD86 (Figure 4F) as compared with the other groups. However, there was no difference in Ly6G (Figure 4G) and CD14 (Figure 4H) expression as comparing with the other groups.

**Figure 5:**
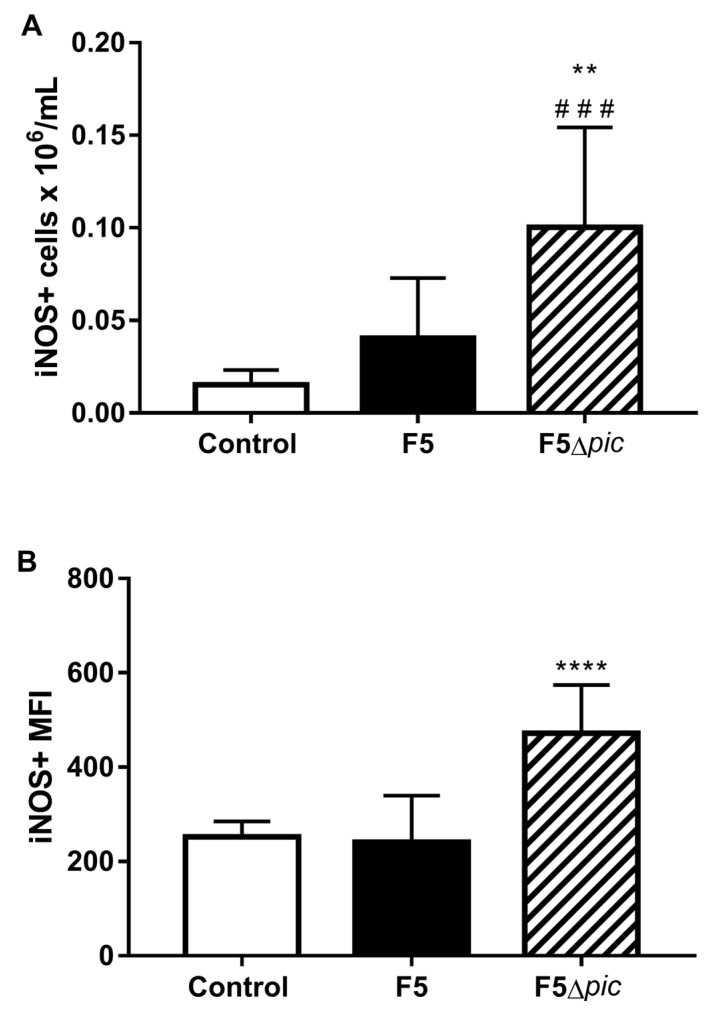
Production of inducible nitric oxide synthase (iNOS) enzyme on peritoneal cavity. After cell counts of the peritoneal cavity, 10^6^ cells/mL were resuspended in specific buffer, transferred to a 96-well U-bottom plate, labeled with specific antibodies, and incubated at 4 °C for 15 min. (**A**) Total number of leukocytes from the peritoneal cavity expressing iNOS; (**B**) the median fluorescence intensity (MFI) of the iNOS. The results represent the mean ± S.D. of 6 animals per group. Data were analyzed using ANOVA, followed by Tukey’s multiple test. ** *p* < 0.01 as compared with the F5 group, **^###^** *p* < 0.001 as compared with the control group, **** *p* < 0.0001 as compared with the other groups.

The number of peritoneal leukocytes expressing iNOS was higher in the mutant group than in the other groups (Figure 5A). The inducible nitric oxide synthase (iNOS) expression in the F5Δ*pic* group was also superior in relation to the other groups, while the expression of this enzyme in the F5 group did not differ from the control group (Figure 5B).

### 2.6. The Expression of Costimulatory Molecules in CD4 T Lymphocytes in the Spleen is Compromised

**Figure 6:**
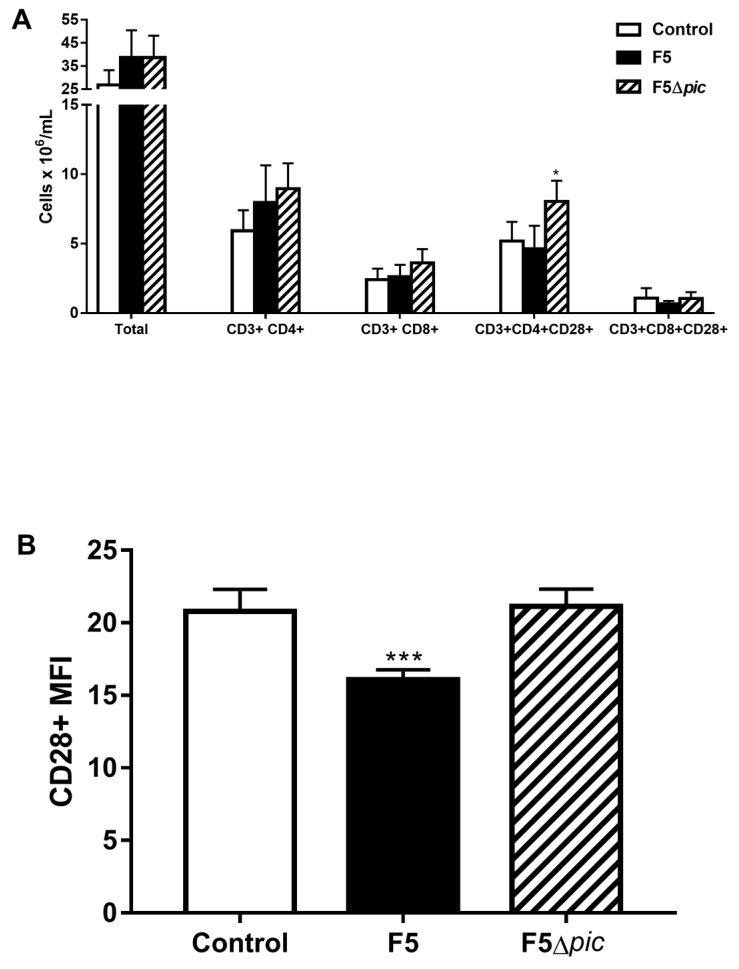
Expression of costimulatory molecules by lymphocytes from spleen. After spleen cell counts, 10^6^ cells/mL were resuspended in a specific buffer, transferred to a 96-well U-bottom plate, labeled with specific antibodies, and incubated at 4 °C for 15 min. The antibodies used and their respective conjugates were anti-CD3 conjugated to FITC, anti-CD28 conjugated to PE, and anti-CD4 conjugated to PerCP. (**A**) Total number, TCD4 and TCD8 cells, and activated T lymphocytes (expressing CD28) from spleen; (**B**) median fluorescence intensity (MFI) of the CD28 molecule in TCD4 lymphocytes from the spleen. The results represent the mean ± S.D. of 6 animals per group. Data were analyzed using ANOVA, followed by Tukey’s multiple test. * *p* < 0.05 as compared with the other groups, *** *p* < 0.001 as compared with the other groups.

Immunophenotyping tests showed that infection by Pic-producing bacteria did not alter the total number of lymphocytes from the spleen, but the number of helper T cells expressing CD28 was higher in the mutant than other groups (Figure 6A). In addition, the F5 group also had reduced expression of the CD28 costimulatory molecule in TCD4 lymphocytes (Figure 6B).

### 2.7. Pic-Producing E. coli Promotes Exacerbated Increase of Inflammatory Mediators in Serum and Peritoneum

**Figure 7:**
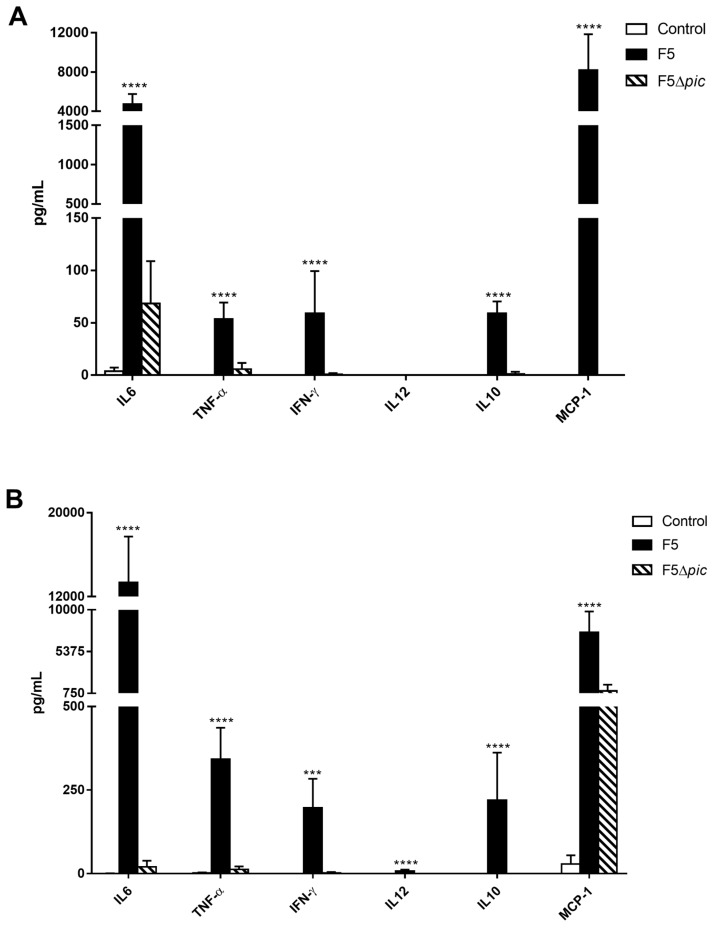
Cytokines production in the animals submitted to intraperitoneal inoculation of bacteria or apyrogenic water. Twelve hours after induction of sepsis by peritoneal inoculation of bacteria, animal sera were obtained and used for cytokine (IL-6, IL-10, TNF-α, IFN-γ, and IL-12) and chemokine MCP-1 dosage. Quantification of these was performed by flow cytometry using the CBA kit. (**A**) Quantification of cytokines in the peritoneal fluid; (**B**) quantification of cytokines in serum. The results represent the mean ± S.D. of 6 animals per group. Data were analyzed using ANOVA, followed by Tukey’s multiple test. *** *p* < 0.001 as compared with the other groups, **** *p* < 0.0001 as compared with the other groups.

Cytokine dosing from peritoneal lavage samples from animals showed a significant production of cytokines in the F5 group, mainly IL-12 (Figure 7A). Both proinflammatory cytokines (IFN-γ, TNF-α, IL-6, and IL-12) and chemokine (MCP-1), and anti-inflammatory cytokine (IL-10) were detected on serum from the F5 group (Figure 7B), with IL-6 and MCP-1 presenting the highest values.

**Figure 8:**
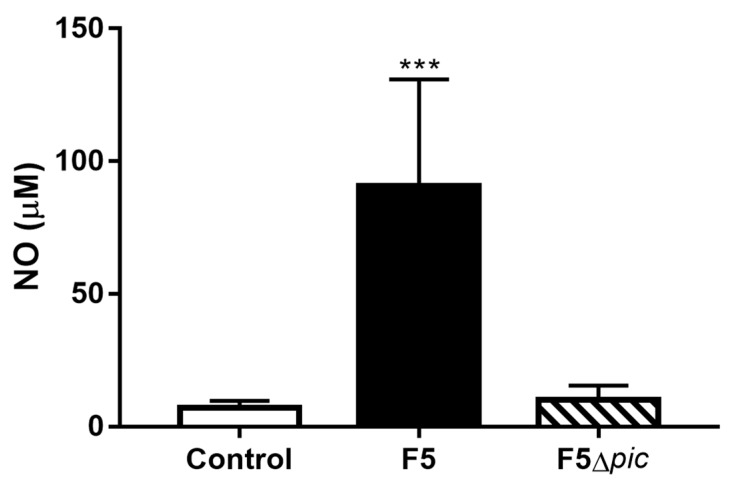
Nitric oxide production in the blood from animals submitted to intraperitoneal inoculation of bacteria or apyrogenic water. Twelve hours after the peritoneal inoculation of bacteria, the animals already anesthetized were submitted to retro-orbital puncture for collection of the blood destined for NO serum measurement via nitrite dosing. The results represent the mean ± S.D. of 6 animals per group. Data were analyzed using ANOVA, followed by Tukey’s multiple test. *** *p* < 0.001 as compared with the other groups.

NO in serum was also produced more strongly by animals infected with Pic-producing bacteria (Figure 8). However, the production of H_2_O_2_ in the peritoneal cavity showed no significant difference between the groups.

## 3. Discussion

In this study, we investigated the role of a Pic-producing *E. coli* in a murine model of sepsis. After intraperitoneal infection of Swiss mice with Pic-producing *E. coli* (F5), F5∆*pic,* or apyrogenic water, blood cultures showed that only F5 was able to survive in the bloodstream. These data indicate that Pic is essential for the survival of the bacterium in the circulation, since the mutant was not able to do it.

Henderson et al. demonstrated that Pic promotes serum resistance in an in vitro model [21]. Its resistance mechanism was elucidated by our group, when we showed that Pic promoted the direct cleavage of key molecules from the three pathways of the complement system, inactivating one of the major mechanisms of immune system [31]. On the basis of this, we believe that *E. coli* F5 survived in the bloodstream due to complement cleavage.

In fact, analysis of complement activity is an important instrument to evaluate the mechanism of action by which Pic provides immune evasion in the bloodstream. Thus, in vivo assays using complement system molecules are currently in progress in our laboratories, in order to better understand the role of Pic in the context of sepsis. However, the sepsis process cannot be exclusive to complement system inactivation, since Pic can cleave other biological substrates, which could have also contributed to the sepsis and death of animals.

The survival of bacteria in the serum increases their potential of dissemination throughout the body, favoring their permanence in several organs, such as spleen, liver, lung, and possibly other organs, and thus promotes widespread inflammation that can culminate in the death of animals in up to 24 h.

Histological samples from the spleen, liver, and kidneys of the animals showed hemorrhage, cellular infiltrate, and edema in the organs of the animals of groups F5 and F5Δ*pic*. These changes were certainly caused by the presence of bacteria in the tissues, regardless of the presence of Pic. Previous studies have pointed out that Pic has no cytopathic effect [29] and is not able to induce tissue damage. Thus, probably other virulence factors present in both bacteria can be associated with damage.

The F5 group presented a higher number of bacteria in the peritoneum 12 h after infection as compared with the F5Δ*pic* group. This result points to a failure to eliminate the Pic-producing bacteria, which could be due to a failure in the production of microbicidal agents, as well as a failure in leukocyte activation in the initial infection focus. In addition, Pic-producing bacteria compromised the expression of several cell surface molecules in leukocytes from peritoneal cavity of animals belong to the F5 group, such as CD3, CD4, CD8, CD19, CD80, and CD86. Low expression of all these molecules certainly resulted in a reduced leukocyte responsiveness and, consequently, impaired the immune response and bacterial proliferation control.

It is worth mentioning that the animals infected with *E. coli* F5 could have presented a reduction of costimulatory molecules due to the cleavage of these molecules by Pic. Ruiz-Perez et al. [28] and Ayala-Lujan et al. [37] have already demonstrated the proteolytic action of Pic on leukocyte superficial glycoproteins, thus, interfering in several mechanisms of the immune response such as activation, migration, and signaling of leukocytes.

The production of microbicidal agents also seems to have been affected. Although the production of hydrogen peroxide in the peritoneal cavity showed no significant difference between the groups, the expression of iNOS in the F5 group was similar to the control group, indicating an impairment in the of nitric oxide production by this group in the infectious focus, whereas the expression of this enzyme was increased in the F5Δ*pic* group.

To date, other studies have already demonstrated the importance of NO synthesized via iNOS in the control of sepsis infection, where iNOS deficient mice failed to contain replication of *Listeria monocytogenes* in vivo [38]. In another study, iNOS-deficient mice submitted to lethal and sublethal sepsis induced by cecum ligation and perforation (CLP) suffered a high lethality rate, attributed to the lack of antimicrobial activity of iNOS-deficient neutrophils and their inability to produce NO. Infection control failure was also evidenced by the high number of bacteria detected in these iNOS deficient mice [39].

Beyond the cellular responsiveness, the number of leukocytes in the bloodstream also showed differences between groups. The blood cell count showed a low number of leukocytes in the group infected with Pic-producing bacteria, especially lymphocytes. On the one hand, this reduced number of lymphocytes in the bloodstream could be a consequence of the migration of these cells into the peritoneal cavity, since the number of lymphocytes was increased inside peritoneum. However, this migration probably cannot contribute to the resolution of the infection due to the low expression of molecules, which are essential for leukocyte activation, as discussed above.

On the other hand, the infection with F5Δ*pic* caused an increase in the leukocytes total number, especially neutrophils, which were not observed in the F5 group. Neutrophils are essential in the context of the innate immune response, playing a key role during the initial immune response in sepsis to eliminate the microorganisms [40] and a failure in their production or function can impact the ability of the immune system to control the infection [41]. Thus, the decrease of neutrophils in the F5 group could also have contributed to the high mortality found in this group.

Here, the serine protease Pic was still shown to be an important molecule inducing the production of cytokines and a chemokine, since IFN-γ, TNF-α, IL-6, IL-12, IL-10, and MCP-1 were detected in the serum from F5 group, significantly higher than other groups. IL-6 and MCP-1 presented the highest values in both serum and peritoneal lavage of the F5 group. Other studies have also reported a high production of cytokines in the context of sepsis, such as IL-6, as well as the chemokine MCP-1 [42,43]. The exacerbated cytokines production is indicative of poor prognosis in patients with sepsis, being associated with a higher mortality rate. Higher values of IL-6 measured during the period of hospitalization in intensive care units showed a maintenance of the inflammatory stimulus, being associated with the later death of the patients, whereas a decrease of this cytokine was found among surviving patients, showing that an increase in IL-6 levels could be considered a useful early marker in the prediction of mortality among septic patients [44]. High concentrations of MCP-1 have also been related to mortality in sepsis. Some studies have found higher concentrations of MCP-1 in patients who died than the concentrations among survivors [45], and therefore were significantly reduced with sepsis resolution [46].

In addition to the production of cytokines, a high NO production on serum was observed in the F5 group. Although the enzyme iNOS has shown low expression in the F5 group, several cells, such as endothelial cells, can produce nitric oxide and its production can be catalyzed by other isoforms of nitric oxide synthase [47]. Excessive NO production in the course of sepsis promotes peripheral vasodilation, blood pressure lowering, with consequently reduction of blood perfusion and tissue hypoxia, which contributes to multiple organ failure [48,49]. Thus, this could have been an additional factor contributing to the high mortality rate in the F5 group.

In summary, the results obtained in this study showed that a Pic-producing *E. coli* promotes lethal sepsis in mice, up to 12 h after infection. Possibly, this is due to the ability of bacteria to survive in the bloodstream and compromise the expression of several cell surface molecules in leukocytes, as well as induce a high production of inflammatory mediators by the host, leading to death by sepsis.

## 4. Material and Methods

### 4.1. Bacterial Samples

To carry out this work, a Pic-producing *E. coli* (F5), as well as its mutant in *pic* gene (F5Δ*pic*) were used. *E. coli* F5 is an extraintestinal pathogenic *E. coli* (ExPEC) that belongs to the serogroup O6 and phylogenetic group B2 which was isolated from the bloodstream of a hospitalized patient at the Hospital São Paulo (Federal University of São Paulo, SP, Brazil). Several virulence genes were screened in this strain and *pic* was detected, in addition to *fimA*, *ompA*, *ompT*, and α*hlyA*. Production of Pic was confirmed by immunoblotting. Other searched ExPEC-associated genes were not detected by PCR which included: *papA, papC, eha, tsh, afaDE, afaBC III, afaE8, clpG, ehx, cnf1, kpsMT II, kpsMT III, cvaC, sitA, irp2, iroN, sat,* and *vat*.

The F5Δ*pic* strain was constructed using the λ Red system [50]. Specific primers flanked by 50 nucleotides extensions homologous to the regions adjacent to the *pic* gene were used to amplify the chloramphenicol cassette from plasmid pKD3 [20]. The purified PCR product was electroporated into strain F5 containing the λ Red recombinase plasmid pKOBEG [51]. Transformed bacterial cells were plated and grown on LB agar containing apramycin and kanamycin at 37 °C. Deletion of the *pic* was confirmed by PCR and DNA sequencing.

Bacterial strains were aerobically grown in Luria-Bertani (LB) broth at 37 °C for 18 h, unless otherwise stated. Chloramphenicol (20 µg/mL) was used when necessary. All strains were kept in LB broth supplemented with 15% glycerol at –80 °C.

### 4.2. Animals

Thirty-six female Swiss mice, from six- to eight-weeks old, weighing on average 25 g were used. The animals were obtained at the Central animal house of the Federal University of Maranhão (São Luís, Brazil) and maintained at 26 ± 2 °C, 44% to 56% relative humidity, under 12 h light-dark cycles and maintained with free access to sterile food and acidified water. All procedures were assessed and approved by the Committee of ethics in research at the Federal University of Maranhão on 25 August 2015 (Process n°. 23115.006231/2015-20).

### 4.3. Bacteria Intraperitoneal Inoculation

The bacteria intraperitoneal inoculation was performed as previously described [52]. After determination of the appropriate inoculum for the induction of lethal sepsis, which consisted of 200 μL of suspensions containing 10^9^ CFU/mL, the suspensions were inoculated intraperitoneally in the animals.

### 4.4. Experimental Design

The animals were initially divided into three groups (12 animals/group). Control group received injections containing 200 μL of apyrogenic water and the F5 and F5Δ*pic* groups were inoculated with one dose of *E. coli* F5 and its *E. coli* F5Δ*pic* mutant, respectively.

After twelve hours of intraperitoneal inoculation, half of the animals from each group were anesthetized to collect the blood and, then, euthanized to collect samples for the tests described below. The mice were euthanized with an overdose of the anesthetic (150 mg/kg ketamine hydrochloride and 120 mg/kg xylazine hydrochloride), and another half of the animals were maintained alive to evaluate the lifespan. The survival of the animals was evaluated every 12 h until the 5th day after bacteria inoculation [42].

### 4.5. Blood Acquisition

Initially, the animals were anesthetized via an intramuscular injection with a solution of 2% xylazine chloridate (20 mg/kg) and 5% ketamine chloridate (25 mg/kg) in a 2:1 ratio, and submitted to retro-orbital puncture for blood acquisition in order to have the differential cell counting, cytokine, and nitric oxide (NO) measurements, beyond evaluation of the presence of bacteria in the blood, by MacConkey agar culture, a selective medium for Gram-negative bacteria. Blood cell counts were performed by automated method using Bioclin 2.8 Vet (Bioclin, Belo Horizonte, Minas Gerais, Brazil).

### 4.6. Peritoneal Cell Harvesting

Mouse peritoneal cells were harvested by washing the peritoneal cavity with 5 mL sterile ice-cold phosphate buffered solution (PBS). Total cell number was estimated by counting cells in a hemocytometer. Then, spleen, liver, kidneys, and lung were collected and weighed. Fragments of the spleen, liver, and lung were weighed and plated on MacConkey agar for determination of colony forming units (CFUs), while fragments of the spleen, liver, and kidneys were used for histopathological analysis.

### 4.7. Colony Forming Units (CFU) Determination

Serial dilutions from the blood, peritoneal lavage, and macerated organs (spleen, liver, and lung) using PBS, were plated on MacConkey agar to evaluate the presence of bacteria. For culture of blood and peritoneal lavage, aliquots of 10 μL and 100 μL, respectively, were diluted in sterile PBS, followed by serial dilutions to the concentration of 10^−5^. For evaluation of the bacteria in the organs, fragments were weighed, ground, and homogenized in sterile PBS and serial dilutions up to the concentration of 10^−5^ were seeded on MacConkey agar. CFUs were counted after overnight incubation at 37 °C, and the results were expressed as Log of CFU/mL.

### 4.8. Histopathological Evaluation

Fragments of liver, spleen, and kidney were removed, weighed and fixed in 10% formaldehyde for 24 h, dehydrated in alcohol and embedded in paraffin. Slides with 5 mm tissue fragments were stained in hematoxylin and eosin for histopathological analysis of the following parameters: hemorrhage, cellular infiltrate, necrosis, and edema. The analysis was determined in double blind test from the following classification: 0 = absent, 1 = slight, 2 = moderate, and 3 = intense.

### 4.9. Hydrogen Peroxide Production

To evaluate H_2_O_2_ release, a horseradish peroxidase-dependent phenol red oxidation micro assay was used [53,54]. In this assay two million peritoneal cells were suspended in 1 mL freshly prepared phenol red solution that consisted of ice-cold Dulbecco’s PBS containing 5.5 mM dextrose, 0.56 mM phenol red (Sigma), and 8.5 U/mL horseradish peroxidase type II (Sigma). One hundred microliters of the cell suspension were added to each well and incubated in the presence or not of 10 ng phorbol myristate acetate (PMA) (Sigma), for 1 h at 37 °C, in a humid atmosphere containing 5% CO_2_ and 95% air. The plates were centrifuged once at 150 × g for 3 min and the supernatants were collected and transferred to another plate. The reaction was stopped with 10 µL 1N NaOH. The absorbance was measured at 620 nm with a microplate reader (MR 5000, Dynatech Laboratories Inc., Gainesville, VA, USA). Conversion of absorbance to µM H_2_O_2_ was done by comparison to a standard curve obtained with known concentrations of H_2_O_2_ (5 to 40 µM).

### 4.10. Counting of Spleen and Bone Marrow Cells

After euthanasia, the femur and spleen were removed. The femur was perfused with 1 mL of PBS to obtain bone marrow cells, and the spleen was removed, crushed with 5 mL PBS and passed through a silk sieve. For total cell number counting, 90 μL of each cell suspension was fixed and stained with 10 μL 0.05% crystal violet in 30% acetic acid. The cells were counted using a Neubauer chamber with the aid of an optical microscope at 400x magnification. The percentage of cell subpopulations was calculated based on the count of 100 cells and transformed in absolute number based on the total count.

### 4.11. Immunophenotyping of Spleen and Peritoneum Cells

After cells counting from the peritoneal cavity and spleen, 10^6^ cells/mL of each were resuspended in immunophenotyping specific buffer and transferred to a 96-well U-bottom plate. Then, the cells were labeled with specific antibodies and incubated at 4 °C for 15 min. In the marking of spleen cells, two panels were used with the following fluorochrome-conjugated monoclonal antibodies: FITC-conjugated anti-CD3, anti-CD28 conjugated to PE, and anti-CD4 and anti-CD8 conjugated to PerCP. For the cells of the peritoneum, the 05 marking panels were assembled as follows: anti-CD3 and anti-CD14 conjugated with FITC; anti-CD80, anti-CD86, and anti-CD19 conjugated to PE; and anti-CD4, anti-CD8, anti-iNOS, and anti-Ly6G conjugated to PerCP (Becton Dickinson Biosciences, San Jose, CA, EUA).

### 4.12. Determination of Nitric Oxide (NO) Production

To measure NO seric production, serum aliquots of 200 μL were deproteinized in the presence of 20 μL of 1 M zinc chloride. After, the samples were centrifuged at 14,000 rpm for 10 min at room temperature. Thereafter, the supernatant was collected for nitrite dosage as an indirect way to evaluate the production of nitric oxide [55].

### 4.13. Quantification of Cytokines

Serum and peritoneal lavage from animals were used for cytokine dosing by flow cytometry. The cytometric bead array (CBA) technique was used for the quantification of TNF-α, MCP-1, IL-6, IL-10, IL-12, and IFN-γ [40], using a mouse inflammation cytokine kit (Becton Dickinson Biosciences, San Jose, CA, EUA).

### 4.14. Statistical Analysis

Results were expressed as the mean ± standard deviation. Statistical analyzes were performed using ANOVA, followed by Tukey’s multiple test, Student’s *t*-test or Kruskal–Wallis and Mann–Whitney tests when the data normality assumption was not satisfied, using Graph Pad Prism software, version 7.0. The differences were considered to be significant when *p* ≤ 0.05. The lifespan of the mice was demonstrated using the Kaplan–Meier curve, and the log-rank statistical test was applied to compare the curves.

## Figures and Tables

**Table 1:** Histopathological evaluation of kidneys, spleen, and liver from animals submitted to intraperitoneal inoculation of bacteria.

Organ	Group	Hemorrhage	Infiltrated	Necrosis	Edema
**Kidneys**	Control	0	0	0	0
	F5	1.0 ± 0.6	1.4 ± 0.5	0	1.6 ± 0.5
	F5∆*pic*	0.16 ± 0.3	1.33 ± 0.4	0	0.83 ± 0.7
**Spleen**	Control	0	0	0	0
	F5	1.2 ± 1.2	2.25 ± 0.4	0	1.2 ± 1.0
	F5∆*pic*	0.8 ± 0.7	2.0 ± 0.6	0	1.8 ± 1.2
**Liver**	Control	0	0	0	0
	F5	0	1.0 ± 0.0	0	0.2 ± 0.4
	F5∆*pic*	0	0.83 ± 0.7	0	0.66 ± 0.7

Twelve hours after peritoneal inoculation of bacteria, liver, spleen, and kidney of the animals were removed, weighed, and fixed in 10% formaldehyde for 24 h, dehydrated in alcohol and embedded in paraffin. Histopathological analysis was performed using an optical microscope. *n* = 6 animals/group; The results are expressed as mean ± SD of the scores. 0, absent; 1, mild; 2, moderate; and 3, intense.

**Table 2:** Spleen, bone marrow, and peritoneal cells counting of animals submitted to intraperitoneal inoculation of bacteria or apyrogenic water.

Cells Counting	Control	*E. coli* F5	*E. coli* F5∆*pic*
Spleen (× 10^6^/mL)	26.86 ± 6.40	38.68 ± 11.84	38.68 ± 9.50
Bone marrow (× 10^6^/mL)	4.74 ± 1.74	4.48 ± 1.77	5.77 ± 1.02
Peritoneum (× 10^6^/mL)	1.568 ± 0.26	0.99 ± 0.43	1.332 ± 0.68

Twelve hours after induction of sepsis, spleen, bone marrow, and peritoneal cells were obtained, stained and counted with the aid of a light-optical microscope. The results represent the mean ± S.D. of 6 animals per group. Data were analyzed using ANOVA.
